# Supplementary material for: An ex vivo animal model to study the effect of transverse mechanical loading on skeletal muscle
Source: Commun Biol. 2024 Mar 9;7:302. doi: 10.1038/s42003-024-05994-0 (PMC10925026; doi:10.1038/s42003-024-05994-0)
Supplement: Supplementary file 2 — Reporting Summary [file 42003_2024_5994_MOESM2_ESM.pdf]

nature portfolio

Corresponding author(s): Arjan Buis

Last updated by author(s): Feb 3, 2024

Reporting Summary

Nature Portfolio wishes to improve the reproducibility of the work that we publish. This form provides structure for consistency and transparency in reporting. For further information on Nature Portfolio policies, see our Editorial Policies and the Editorial Policy Checklist.

Statistics

For all statistical analyses, confirm that the following items are present in the figure legend, table legend, main text, or Methods section.

n/a	Confirmed	
☐	☒	The exact sample size (*n*) for each experimental group/condition, given as a discrete number and unit of measurement
☐	☒	A statement on whether measurements were taken from distinct samples or whether the same sample was measured repeatedly
☐	☒	The statistical test(s) used AND whether they are one- or two-sided<br>*Only common tests should be described solely by name; describe more complex techniques in the Methods section.*
☒	☐	A description of all covariates tested
☒	☐	A description of any assumptions or corrections, such as tests of normality and adjustment for multiple comparisons
☐	☒	A full description of the statistical parameters including central tendency (e.g. means) or other basic estimates (e.g. regression coefficient) AND variation (e.g. standard deviation) or associated estimates of uncertainty (e.g. confidence intervals)
☒	☐	For null hypothesis testing, the test statistic (e.g. *F*, *t*, *r*) with confidence intervals, effect sizes, degrees of freedom and *P* value noted<br>*Give P values as exact values whenever suitable.*
☒	☐	For Bayesian analysis, information on the choice of priors and Markov chain Monte Carlo settings
☒	☐	For hierarchical and complex designs, identification of the appropriate level for tests and full reporting of outcomes
☒	☐	Estimates of effect sizes (e.g. Cohen's *d*, Pearson's *r*), indicating how they were calculated

Our web collection on statistics for biologists contains articles on many of the points above.

Software and code

Policy information about availability of computer code

Data collection	Motic images plus 3.0 software, Leica Application Suite X V.3.1.5.16308
Data analysis	ANSYS Workbench 2021 R2, Zen 3.1 (Carl Zeiss) , FIJI/ImageJ (Vers. 1.53s, NIH), Cellpose 1.0, Minitab (Vers. 19.2020.1)

For manuscripts utilizing custom algorithms or software that are central to the research but not yet described in published literature, software must be made available to editors and reviewers. We strongly encourage code deposition in a community repository (e.g. GitHub). See the Nature Portfolio guidelines for submitting code & software for further information.

Data

Policy information about availability of data

All manuscripts must include a data availability statement. This statement should provide the following information, where applicable:

- Accession codes, unique identifiers, or web links for publicly available datasets
- A description of any restrictions on data availability
- For clinical datasets or third party data, please ensure that the statement adheres to our policy

The authors declare that the data supporting the findings of this study are available within the paper. Should any raw data files be needed in another format they are available from the corresponding author upon reasonable request.

Research involving human participants, their data, or biological material

Policy information about studies with human participants or human data. See also policy information about sex, gender (identity/presentation), and sexual orientation and race, ethnicity and racism.

Reporting on sex and gender	NA
Reporting on race, ethnicity, or other socially relevant groupings	NA
Population characteristics	NA
Recruitment	NA
Ethics oversight	NA

Note that full information on the approval of the study protocol must also be provided in the manuscript.

Field-specific reporting

Please select the one below that is the best fit for your research. If you are not sure, read the appropriate sections before making your selection.

☒ Life sciences ☐ Behavioural & social sciences ☐ Ecological, evolutionary & environmental sciences

For a reference copy of the document with all sections, see nature.com/documents/nr-reporting-summary-flat.pdf

Life sciences study design

All studies must disclose on these points even when the disclosure is negative.

Sample size	Sample sizes for the histology-based feasibility study of n >= 5 were chosen based on similar in vivo experiments for pressure injury research (Linder-Ganz et al. 2006). The sample size of n = 1 muscles for each loading regime within the following fluorescence study (overall sample size of n = 12) was set to minimise animal usage. This is also congruent with other animal studies that have reportedly low sample sizes , i.e. by Kosiak et al. (1961) and Bosboom et al. (2003). Despite the low sample size number, similarities of the cell death effects to other studies as described in the paper support our newly developed approach.
Data exclusions	One outlier was observed at 90 min loading with 21.1 kPa. The sample was classified as “no damage” despite the compressive stress being above the proposed threshold function. This might be related to human error as the staining procedure for this sample deviated from the protocol followed for the other samples, i.e. the sample was not “flipped” to ensure sufficient contact between the sample and the staining solution. Another potential explanation is biological variability. However, with only one sample analysed for each load-time-combination to minimise animal usage, no definitive conclusion could be drawn. With only few data points available, the outlier was excluded from the threshold curve analysis to avoid skewed results.
Replication	The reproducibility of experimental setup was assessed through repeated testing of tissue samples with a sample size of n >= 5. Similar results were observed across the different intervention groups, including direct fixation, MOPS storage, and mechanical indentation. This supports the reproducibility of the experiments.
Randomization	Samples for the histology-based feasibility study were randomly allocated. Because of the restricted time frame of 3.5h for the overall experiments, the samples for the fluorescence study were assigned based on a pre-developed schedule. This ensured that all muscles from an animal could be used for testing.
Blinding	For the histology-based feasibility study, the same investigator performed the experiments as well as the qualitative analysis without blinding. The analysis and results were repeated and confirmed by a second, blinded investigator. For the fluorescence study, blinding was not necessary as a pre-defined quantitative analysis workflow was followed to assess the extent of cellular damage.

Reporting for specific materials, systems and methods

We require information from authors about some types of materials, experimental systems and methods used in many studies. Here, indicate whether each material, system or method listed is relevant to your study. If you are not sure if a list item applies to your research, read the appropriate section before selecting a response.

Materials & experimental systems

n/a	Involved in the study
☒	☐ Antibodies
☒	☐ Eukaryotic cell lines
☒	☐ Palaeontology and archaeology
☐	☒ Animals and other organisms
☒	☐ Clinical data
☒	☐ Dual use research of concern
☒	☐ Plants

Methods

n/a	Involved in the study
☒	☐ ChIP-seq
☒	☐ Flow cytometry
☒	☐ MRI-based neuroimaging

Animals and other research organisms

Policy information about studies involving animals; ARRIVE guidelines recommended for reporting animal research, and Sex and Gender in Research

Laboratory animals	6 – 7 weeks old male Sprague-Dawley rats
Wild animals	No wild animals were involved in this study.
Reporting on sex	Only male animals were used for this study.
Field-collected samples	No field-collected samples were involved in this study.
Ethics oversight	All experiments were in line with the ethical guidelines of the Biological Procedures Unit at the University of Strathclyde. As the the tissue was taken from rats that were culled without previous intervention, no further ethical approval was necessary.

Note that full information on the approval of the study protocol must also be provided in the manuscript.
